# Impact of Bioconjugation on Structure and Function of Antibodies for Use in Immunoassay by Hydrogen-Deuterium Exchange Mass Spectrometry

**DOI:** 10.3389/fmolb.2022.866843

**Published:** 2022-07-07

**Authors:** Luise Luckau, Kate Groves, Chris Blencowe, Sam Scrimshaw, Alastair Dent, Milena Quaglia

**Affiliations:** ^1^ National Measurement Laboratory at LGC, Teddington, United Kingdom; ^2^ Fleet Bioprocessing Ltd., Hartley Wintney, United Kingdom

**Keywords:** hydrogen-deuterium exchange mass spectrometry, antibody conjugate, immunoassay, lysine conjugation, cysteine conjugation, structure-function analysis

## Abstract

Monoclonal antibodies (mAbs) are widely used as analytical components in immunoassays to detect target molecules in applications such as clinical diagnostics, food analysis and drug discovery. Functional groups are often conjugated to lysine or cysteine residues to aid immobilization of mAbs or to enable their detection in an antibody antigen complex. Good assay performance depends on the affinity and specificity of the mAbs for the antigen. The conjugation reaction however can cause higher order structural (HOS) changes and ultimately affect the assay performance. In this study, four differently conjugated mAbs were selected as model systems and characterized by mass spectrometry. Particularly, intact protein analysis by liquid-chromatography mass-spectrometry (LC-MS) was performed to determine the amount and distribution of conjugation. Hydrogen deuterium exchange mass spectrometry (HDX-MS) experiments were carried out for the structural characterization of the conjugated mAbs. Immunoassay experiments were performed to monitor the effects of conjugation on the binding properties of the antibodies selected. Good agreement between the mass spectrometry and binding experiment results was found. Particularly, it was noted that the overall structural flexibility of the antibodies increases upon cysteine conjugation and decreases for lysine conjugation. The conjugation of mAbs with bulky functional groups tends to decrease the deuterium uptake kinetics due to induced steric effects. Overall, this study shows correlations between conjugation, structure and function of immunoassay antibodies and the benefits of mass spectrometry to improve understanding of the conjugation reaction and provide insights that can predict immunoassay performance.

## Introduction

Immunoassays are bioanalytical methods that use the specificity of an antibody to detect and quantify target molecules in complex matrices. They are known for their high sensitivity and specificity and are widely used in clinical diagnostics, drug discovery, food and environmental testing (Xiao and Lin 2015; Di Nardo et al., 2021). Various immunoassay designs are in use including competitive and immunometric (“sandwich”) formats. The format selected depends on the analyte, the available reagents and the dynamic range required for the particular assay (Cox et al., 2019). Sandwich assays have been reported to be more sensitive and robust and are therefore most commonly used (Cox et al., 2019). In sandwich assays, antigen-capture antibodies are often biotin-conjugated to enable immobilization onto streptavidin-coated assay surfaces. The detection of the antibody-antigen (Ab-Ag) complex is achieved by a second antigen-specific antibody, which is typically conjugated with reporter molecules such as fluorophores or enzymes that, in the presence of an Ab-Ag complex, generate a measurable response. Method development and validation is essential to provide a good and reliable assay performance in terms of sensitivity and specificity. As part of this, the characterization of the conjugated mAb has an important role, as for example the amount and location of conjugation can have an impact on structure and function of the mAb and consequently its binding affinity to the antigen of interest. To the best of our knowledge, the steric effects of conjugation on antibody binding and any changes in the antibody higher order structure (HOS) due to conjugation have yet to be investigated. Mass spectrometry is a powerful tool for characterization of mAb conjugates as it can provide both detailed information on the level of conjugation and any mAb structural changes induced by the conjugation (Huang and Chen, 2016; Leurs et al., 2017; Zhu et al., 2020). Particularly, top-down high-resolution intact protein analysis by liquid chromatography - mass spectrometry (LC-MS) can provide information on the amount and distribution of conjugation between heavy chain (HC) and light chain (LC) subunits (Wakankar et al., 2011; Zhu et al., 2020). Hydrogen-deuterium exchange mass spectrometry (HDX-MS) is a powerful technique for the structural characterization of large proteins such as mAbs in solution and for definition of any locally induced structural changes (such as those caused by conjugation) (Konermann et al., 2014; Zhang et al., 2014; Groves et al., 2020). HDX-MS has shown great potential in characterizing protein-protein interactions, providing useful information on the antigen binding site of mAbs (paratope mapping) and the binding sites on the antigen (epitope mapping) (Puchades et al., 2019; Sun et al., 2021; Zhu et al., 2021). During HDX-MS experiments, structural changes or protein-protein interactions result in variations in the exposure of the amide protein backbone towards surrounding solvent when incubated in deuterated water, affecting the ability of the amide protons to exchange with deuterions in the solvent. In a typical bottom-up HDX-MS workflow, changes in HDX rates of proteins or protein complexes are monitored at different incubation times. After incubation, the reaction is quenched at low pH and the proteins of interest are proteolytically digested using acid tolerant proteases, most typically pepsin. In order to obtain valuable insights into structural differences at the residue level, in particular to define protein-protein interaction sites, a high level of protein coverage and sequence overlapping proteolytic peptides (redundancy) is required. Due to the increased complexity of peptide mixtures when analyzing mAbs ( ∼ 148 kDa) and Ab-Ag complexes (>148 kDa), peptide identification and data analysis can become more challenging due to co-eluting peptides and interferences. To overcome this, ion mobility spectrometry (IMS) can be used as an orthogonal dimension of peptide separation to the reverse phase separation generally used for HDX-MS workflows, by also separating peptides according to their size and charge as gas phase ions. Additionally, it has been shown that peptide identification of more complex biological samples can be significantly increased when the data independent acquisition (DIA) approach, MS^E^, is combined with IMS (HDMS^E^) and furthermore, when optimized precursor collision energies are applied (UDMS^E^) (Cryar et al., 2017).

In this study, intact protein analysis by LC-MS and HDX-IM-MS with the UDMS^E^ approach were applied to characterize conjugated antibodies. A panel of commercially available mAbs (Humira (Adalimumab), Opdivo (Nivolumab), Xolair (Omalizumab) and Herceptin (Trastuzumab)) were selected to undergo conjugation with biotin and fluorescein at lysine (Lys or K) or cysteine (Cys or C) residues using different linker reagents and chemistries. The selection of the most appropriate combination “conjugation:antibody:antigen” for MS analysis was performed by immunoassay. Four conjugates were selected: fluorescein-isothiocyanate (FITC) conjugated Adalimumab at Lys residues (Adalimumab-Lys), biotinylated Nivolumab *via* an N-hydroxy succinimide (NHS) activated ester reacting at Lys residues (Nivolumab-Lys), fluorescein labelled Omalizumab using fluorescein-5-malimide at Cys residues (Omalizumab-Cys) and biotinylated Trastuzumab at Cys residues using a dibromomaleimide (DBM) linker (Trastuzumab-Cys). These conjugates will be referred in this paper as Adalimumab-Lys, Nivolumab-Lys, Omalizumab-Cys and Trastuzumab-Cys respectively indicating the type of mAb and the conjugation at Cys or Lys residues. For the structural characterization of the changes induced upon conjugation of the Omalizumab antibody and Trastuzumab antibody, native and conjugated forms were directly compared by HDX-MS. For the Adalimumab-Lys and Nivolumab-Lys, a more complex experimental design was applied to simultaneously define structural differences between native and conjugated mAbs at the antigen binding site (paratope), which consists of a set of three complementarity-determining regions (CDR) in both the heavy (HC) and light chain (LC), respectively defined as CDR-H1, -H2, -H3, CDR-L1, -L2, -L3. For this purpose, the native mAbs were analyzed and compared to the native and conjugated mAb in complex with their respective antigens: tumor-necrosis factor α (TNFα) for Adalimumab and the programmed cell death protein 1 (PD-1) for Nivolumab.

## Materials and Methods

### Materials

Chemicals such as urea, dithiothreitol (DTT), HABA/Avidin reagent and deuterium oxide were purchased from Sigma-Aldrich (Gillingham, United Kingdom). Potassium phosphate (monobasic, dibasic), formic acid, tris(2-carboxy-ethyl)phosphine (TCEP), biotin-XX SE, biotin-PEG_2_-malimide and FITC were purchased from Fisher Scientific (Loughborough, United Kingdom). Fluorescein-5-maleimide was purchased from TCI (Tokyo, Japan). Trastuzumab, Adalimumab, Nivolumab and Omalizumab were purchased from Medizone (Oberhaching, Germany). All native and conjugated mAbs were provided as solutions in 100 mM potassium phosphate buffer pH 7.5 by Fleet Bioprocessing: 34 µM of Omalizumab, 34 µM Adalimumab, 34 µM Nivolumab, 16 µM of Trastuzumab, 20 µM Adalimumab-Lys, 20 µM Nivolumab-Lys, 20 µM Omalizumab-Cys and 15 µM Trastuzumab-Cys. The recombinant human proteins TNFα (Fisher Scientific, Loughborough, United Kingdom) and PD-1 (Sino Biological, Frankfurt, Germany) were solubilized at 1 g/L (57.5 µM) in water. A tetrapeptide (PPPI) was synthesized and purchased from Thermo Fisher Scientific (Loughborough, United Kingdom). The peptide standard glu-1-fibrinopeptide B was purchased from Sigma-Aldrich (Gillingham, United Kingdom). Optigrade HPLC Special Grade acetonitrile and ultra-pure water (18 MΩ cm^−1^) were used. 96 well polystyrene microtitre plates were purchased from Greiner Bio-One (Stonehouse, United Kingdom). Sure Blue Reserve TMB substrate solution was purchased from Insight Biotechnology (Wembley, United Kingdom). Gyrolab XP, Bioaffy CD1000 and Rexxip buffers were purchased from Gyros Protein Technologies AB (Uppsala, Sweden). Anti-Fc, anti-nivolumab, anti-trastuzumab, anti-omalizumab and anti-adalimumab were purchased from Bio Rad (Watford, United Kingdom).

### Conjugation of Biotin and Fluorescein Labels

The preparation of mAb conjugates was carried out using well-established literature methods and is described in supporting information S1-1. Trastuzumab-Cys was conjugated by mild TCEP reduction and reaction with dibromomaleimide-biotin; the aim was to conjugate a single biotin across one reduced inter-chain disulfide residue. Omalizumab-Cys was conjugated by forcing conditions using TCEP reduction and reaction with fluorescein-maleimide aiming for conjugation to all reduced inter-chain disulfide residues. Adalimumab-Lys and Nivolumab-Lys were conjugated with FITC and biotin-XX-NHS respectively, both aiming for high incorporations of label.

### Intact Protein Analysis by LC-MS

For the intact protein analysis of native and conjugated mAbs, the mAb stock solutions were diluted to a 6 µM mAb concentration in 100 mM Tris (pH 7.4). Deglycosylation of the samples was performed by adding 2 µL peptide:N-glycosidase (PNGase F, 10 ku/mL, Promega, Southampton, United Kingdom) to 18 µL of 6 μM mAb, followed by incubation at 37°C for 24 h. The completion of the deglycosylation of native mAbs was confirmed by LC-MS and deconvoluted masses of glycosylated and deglycosylated mAbs are provided in supporting information S1-2. For the middle-down LC-MS analysis of native and conjugated mAbs, 10 µL DTT (50 mM) was added to 10 µL of the deglycosylated mAb sample and incubated at room temperature for 30 min to separate the HC and LC. For the LC-MS analysis, the deglycosylated and deglycosylated plus reduced mAb samples were diluted to a mAb concentration of 3 μM and 5% acetonitrile.

10.5 pmol of sample was injected onto a MAbPac Reversed Phase HPLC Column (2.1 × 100 mm, 4 μm, Thermo Fisher Scientific) in a Vanquish UHPLC system (Thermo Fischer Scientific, Bremen, Germany). Chromatographic separation was achieved using a linear gradient starting from 85% A/15% B to 70% A/30% B over 1 min to 60% A/40% B over 13 min at a flow rate of 250 μL/min and 65°C column temperature. Mobile phases were aqueous, 0.5% formic acid (A), and acetonitrile, 0.5% formic acid (B).

MS experiments were performed using a Q-Exactive Plus Orbitrap instrument with a HESI-II probe source (Thermo Fisher Scientific, Bremen, Germany) in positive nanoelectrospray ionization (nESI) mode using a 320°C source temperature, 3.5 kV capillary voltage, 100 S-lens RF, 25 a. u. sheath gas, 5 a.u. aux gas, 100 eV in-source CID, 200 ms maximum injection time, 3E^6^ AGC target value and 10 microscans. Data were acquired in 35 K resolution mode over a range of 1,000–5,000 m/z (high mass range (HMR) mode). Protein deconvolution was performed using BioPharma finder v 2.0 software (Thermo Fisher Scientific, Waltham, MA) using the ReSpect algorithm. Deconvolution was performed in the 10–180 kDa range, considering 4–60 charge states, a range of 4–10 minimum charge states and a target mass of 147 kDa.

### Sample Preparation for Hydrogen Deuterium Exchange Experiments

Antibodies and proteins were stored separately at 4°C until analysis. All native and conjugated antibodies were diluted to a final protein concentration of 10 µM with 50 mM potassium phosphate buffer, pH 7.4. For the direct comparison between native and conjugated mAbs, two sets of samples were prepared: Omalizumab *v*s. Omalizumab-Cys and Trastuzumab *vs.* Trastuzumab-Cys. The more complex HDX-MS experiment for the structural characterization of Adalimumab-Lys and Nivolumab-Lys included four samples, the native mAb (Adalimumab or Nivolumab), the free antigen (TNFα or PD-1), the native mAb in complex with antigen (Adalimumab:TNFα, ratio 1:1 and Nivolumab:PD-1, ratio 1:2) and the conjugated mAb in complex with antigen (Adalimumab-Lys:TNFα, ratio 1:1 and Nivolumab-Lys:PD-1, ratio 1:2). The peptide PPPI as internal standard was added to each sample at 10 µM.

### HDX–Ion Mobility Mass Spectrometry

Sample handling and mixing steps were performed using a first-generation LEAP PAL system set (LEAP Technologies, Morrisville, NC). 15 µL sample was diluted 10-fold either in 50 mM potassium phosphate buffer, pH 7.4 for the generation of peptide maps or in 50 mM potassium phosphate buffer (pH 7.0) prepared in D_2_O for exchange experiments. The HDX time course for each HDX-MS data set is summarized in supporting information S2. Each exchange time point was run in triplicate and quenched by a 2-fold dilution with 50 µL of 1 M TCEP, 8 M Urea in 100 mM potassium phosphate buffer (pH 2.5) at 4°C for 10 min. 95 µL of quenched sample was injected onto a refrigerated nano-ACQUITY UPLC system with HDX technology (Waters, Milford, MA) for on-line pepsin digestion and chromatographic separation. Protein digestion was performed on an Enzymate BEH pepsin column (300 Å, 5 μm, 2.1 × 30 mm, Waters, Milford, MA) at a flow rate of 70 μL/min (0.5% v/v formic acid) and a pressure of 4,800 psi. The digestion pressure was held by using a PEEK restrictor placed after the trap column. Digestion temperature was maintained at 15°C for Adalimumab, Trastuzumab, Nivolumab and at 20°C for Omalizumab. After digestion, the proteolytic peptides were trapped and desalted for 3.8 min using an ACE C18 guard cartridge (5 μm, 2.1 mm) and chromatographically separated on an ACE Excel 2 Super C18 column (2 μm, 2.1 × 100 mm, Hichrom, Reading, United Kingdom) at a flow rate of 100 μL/min using a 7 min linear gradient from 8% B to 35% B. Mobile phases were aqueous, 0.1% v/v formic acid (A) and acetonitrile, 0.1% v/v formic acid (B).

ESI-IM-MS experiments were performed on a Synapt G2Si Q-TOF-MS instrument (Waters, Milford, MA) in positive nanoelectrospray ionization mode, sensitivity mode, 100°C source temperature, 3 kV capillary voltage, 80 V cone voltage, 80 V source offset and 250°C desolvation temperature.

For the peptide map generation, the instrument was operated in the UDMS^E^ mode, which is MS^E^ with applied ion mobility and optimized collision energies based on measured ion arrival times. Optimized collision energies were from Distler and co-workers (Distler et al., 2014). Deuterium exchange experiments were acquired in IM-MS mode. Data acquisition in both modes UDMS^E^ and IM-MS was carried out over a range of 50–2000 m/z, a scan time of 0.2 s using the same ion mobility settings as follows: 650 m/s wave velocity, 40 V wave amplitude, 190 m/s transfer velocity, 4 V transfer wave amplitude, 450 µsec mobility trapping release time and a wave velocity ramp of 800–500 m/sec. Data was lock mass corrected against *m/z* 785.8426 of glu-1-fibrinopeptide B (100 fmol solution in 50% methanol, 0.1% formic acid), applied post acquisition.

To generate peptide lists, UDMS^E^ data was analyzed with the ProteinLynx Global Server (PLGS) software v3.0.3 (Waters, Milford, MA) against protein sequences of interest. PLGS outputs were imported to DynamX v3.0 (Waters, Milford, MA) to generate peptide maps and analyze HDX-MS data. The 3σ approach was applied as statistical test for significance. Full details of PLGS and DynamX processing conditions and protein amino acid sequences used for database search are listed in the supporting information S1-3 and S1-4. To allow access to the HDX data of this study, the HDX data summary table and the HDX data table are included in the supporting information S2 and S3 respectively as per consensus guidelines (Masson et al., 2019).

### Determination of Labelling of mAb by Colorimetry

Fluorescein incorporations were determined by UV-vis spectrophotometry using an 495 nm extinction coefficient of 150.5 (mg/ml)^−1^ cm^−1^ and 280:495 Rz of 0.34. Biotin incorporations were determined by HABA/Avidin colorimetric assay following the instructions provided by the reagent supplier, using a 500 nm extinction coefficient of 34,000 M^−1^ cm^−1^.

### Anti-Fc and Antigen Binding Affinity Assay

A description of the different immunoassay formats is provided in the supporting information S1-5. A total of seven different assay formats (Fc binding, complex binding, competitive, immunometric, bridging, Fc capture and streptavidin capture) were used to characterize the conjugate performance relative to each other and the unconjugated mAbs.

## Results and Discussion

### Characterization of Conjugated mAbs by LC-MS

Intact protein analysis of mAbs was carried out to elucidate the extent of conjugation, its location (for example heavy chain (HC) *vs* light chain (LC)) and the sample heterogeneity. The results are summarized in Table 1. The deconvoluted mass spectra and the conjugated mAb amount are shown in supporting information S1-6. In this study both mAbs, Omalizumab and Trastuzumab, belong to the immunoglobulin G subtype 1 class (IgG1), typically containing 12 intra-chain and 4 inter-chain disulfide bridges. In particular, inter-chain disulfide bridges are the target for conjugation as they are more prone to reduction and more accessible to conjugation (Adumeau et al., 2016). An advantage of conjugating at the inter-chain disulfide bridges is that it expected to have lower impact on antigen binding due to its distance from the mAb binding site.

**TABLE 1:** Amount of mAb conjugation by intact LC-MS protein analysis and colorimetry.

mAb Conjugates	Conjugation Amount per Intact mAb by LC-MS				Conjugation Amount per Intact mAb by Colorimetry
	total	most abundant			total
Omalizumab-Cys	n.a	n.a			5.9^a^
Trastuzumab-Cys	0–3	0			0.9^b^
Adalimumab-Lys	2–6	2–4			5.2^a^
Nivolumab-Lys	28–42	35–36			16.1^b^

UV-vis spectroscopy^a^ and by HABA/Avidin colorimetric assay^b^.

**FIGURE 1:**
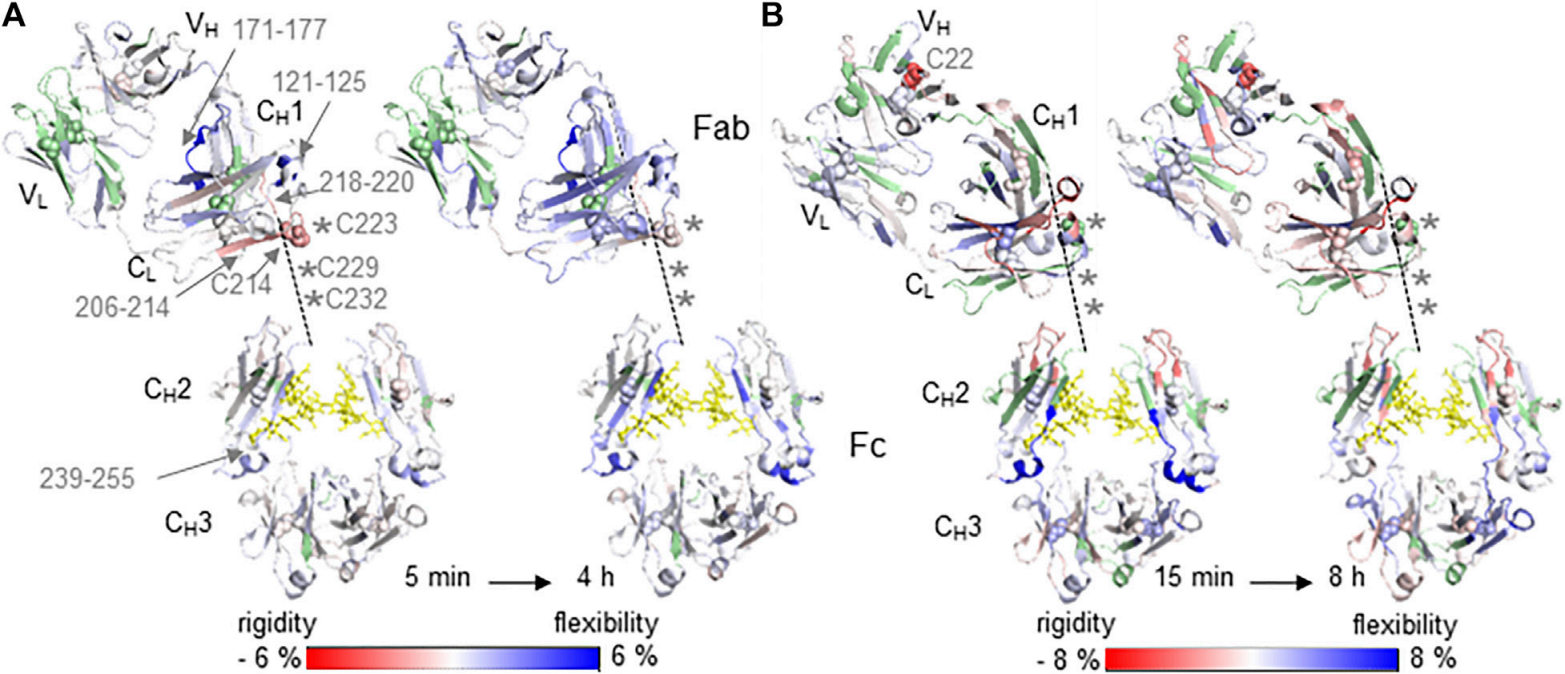
Relative differences in HDX-MS between native (reference state) and cysteine conjugated mAb samples Trastuzumab with DBM-biotin **(A)** and Omalizumab with fluorecein-5-maleimide **(B)** are illustrated on the crystal structure of the generic IgG1 Fc domain (PDB: 5vgp) and the specific Fab domains PDB: 6 mh2 **(A)** and PDB: 2xa8 **(B)**. Increased (blue) and decreased (red) HDX-MS rates as relative changes from the native mAbs are indicated by the color bar and are shown between the lowest and highest time points, from 5 min to 4 h **(A)** and 15 min to 8 h **(B)**. Light chain (LC) domains located in Fab are V_L_ and C_L_. Heavy chain (HC) domains are V_H_ and C_H_1 of Fab and C_H_2/C_H_3 of Fc. The hinge region with inter-chain disulfide bonds is unresolved in crystal 3D structures and is sketched by the dashed line connecting the C_H_1 and C_H_2 of HC. Missing cysteine residues of the HC in the hinge region are indicated as asterisks. Missing sequence information for deuterium uptake kinetics are highlighted in green. Glycans are indicated as yellow sticks and cysteine residues forming disulfide bonds as spheres.

For the Omalizumab antibody, conjugation was achieved by using a commercially available linker fluorescein-5-maleimide. By LC-MS intact protein analysis, no residual intact antibody was detected, and the free HC and LC were observed to contain three and one conjugate molecules respectively (Table 1, supporting information S1-6: Figure 1). This indicated that all eight sulfhydryl groups of the four inter-chain disulfide bonds were conjugated as anticipated and that the structure of Omalizumab-Cys was mainly stabilized through non-covalent interactions between the mAb chains.

**FIGURE 2:**
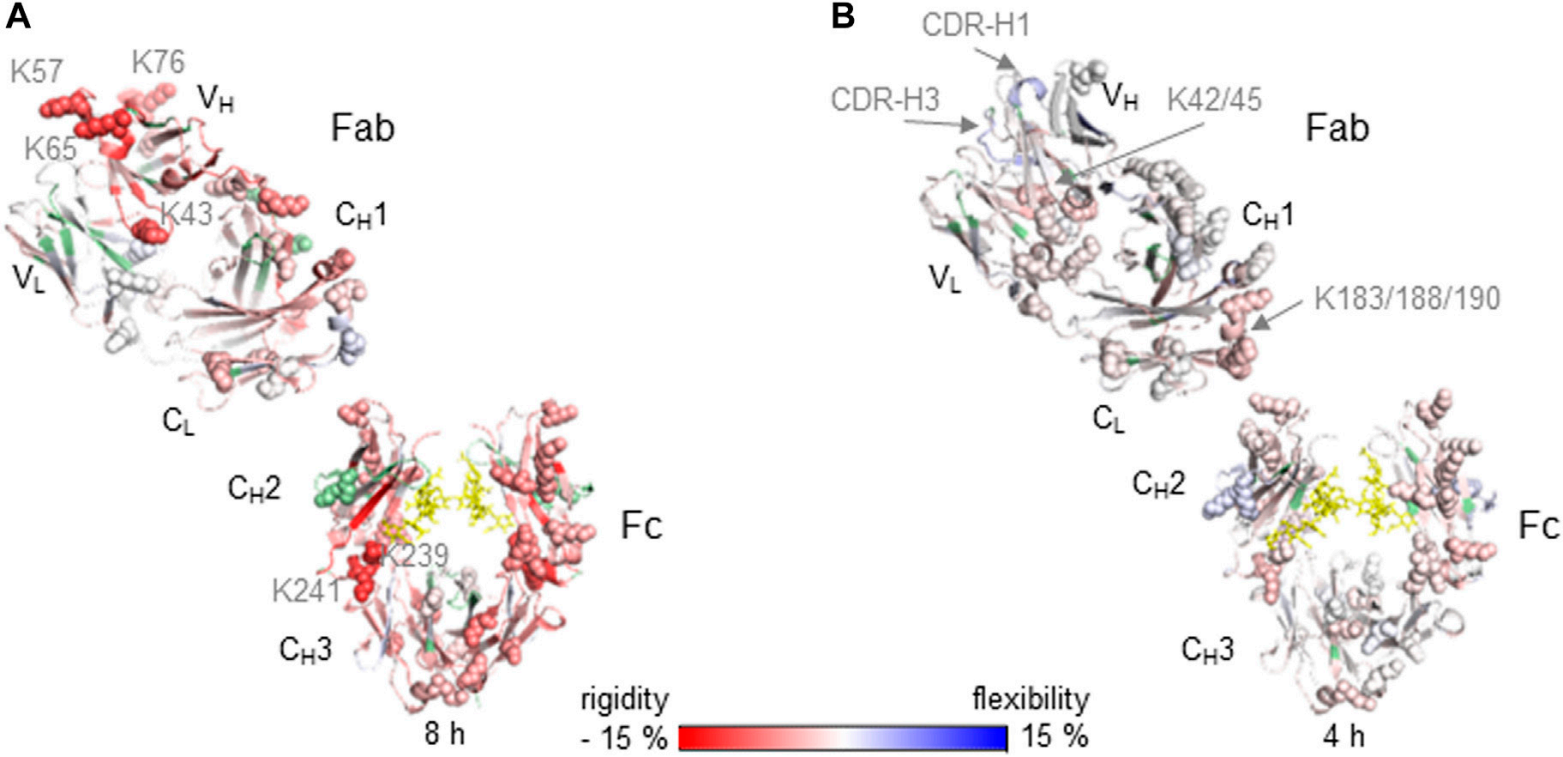
Relative differences in HDX-MS between native (reference state) and lysine conjugated mAb samples are illustrated on the crystal structure for Nivolumab of the generic IgG4 Fc domain (PDB: 4c54) and the Nivolumab specific Fab domain PDB: 5ggq **(A)** and for Adalimumab on the IgG1 Fc domain (PDB: 5vgp) and Adalimumab specific Fab PDB: 3wd5 **(B)**. Increased (blue) and decreased (red) HDX-MS rates as relative changes from the native mAbs are indicated by the color bar and are shown for the highest incubation time 8 h **(A)** and 4 h **(B)**. Light chain (LC) domains located in Fab are V_L_ and C_L_. Heavy chain (HC) domains are V_H_ and C_H_1 of Fab and C_H_2/C_H_3 of Fc. Missing sequence information for deuterium uptake kinetics are highlighted in green. Glycans are indicated as yellow sticks and lysine residues as spheres.

The Trastuzumab was initially conjugated with biotin by using a bifunctional dibromomaleimide (DBM) derived biotin reagent, which was made according to the method of Baker and coworker (Schumacher et al., 2014), and similar reaction conditions as used for Omalizumab-Cys. In this approach, the inter-chain disulfide bonds are “maintained” by cross-linking cysteine bridges via double substitution of the DBM moiety (Badescu et al., 2014). Intact protein analysis of the reduced Trastuzumab conjugate by LC-MS showed that the main mAb populations are the intact antibody and the HC-HC-LC. Only traces of free LC and HC were observed (supporting information S1-6: Figure 2A) indicating that the antibody chains of Trastuzumab-Cys are irreversible cross-linked through the DBM linker. 46% of the intact and HC-HC-LC Trastuzumab-Cys population was unconjugated due to low incorporation efficiency of the biotin-DBM reagent. However, 54% of the intact and HC-HC-LC population was conjugated containing 1-3 biotin molecules/antibody with a relative abundancy of 36.7%, 15.1 and 2.3% respectively (supporting information S1-6: Figure 2 and Tables 1, 2). Because mAb chains as LC and HC are mainly cross-linked using the DBM linker, the conjugation distribution between HC and LC is not clearly determinable. However, the main presence of conjugated intact and HC-HC-LC populations proves that inter-chain disulfide bonds are the target of the conjugation approach.

**TABLE 2:** Relative performance of conjugated mAbs against the native mAbs as measured by different immunoassay formats.

mAb Conjugate	Fc Recognition (%)	Antigen Recognition (%)
Adalimumab-Lys	98.3 (80.4–120.1)^a^	91.0 (74.4–111.2)^b^
Nivolumab-Lys	61.1 (50.0–74.7)^a^	132.5 (108.4–161.9)^c^
Omalizumab-Cys	91.8 (75.1–112.2)^a^	65.7 (53.7–80.3)^b^
Trastuzumab-Cys	67.0 (54.8–81.9)^d^	100.0 (81.8–122.2)^e^

Rel. binding to native mAb: Fc binding^a^, complex binding^b^, competitive^c^, Rel. binding to highest signal of a conjugated mAb panel: Fc capture^d^, streptavidin capture^e^.

As previously described the Adalimumab and Nivolumab antibodies were conjugated at the Lys residues using commercially available FITC (fluorescein-isothiocyanate) and biotin-NHS reagents. Because of the high abundance of Lys residues (86 residues in Adalimumab and 78 residues in Nivolumab) and the statistical nature of Lys-based conjugations, the results from LC-MS (Table 1) showed the presence of a highly heterogeneous mixture of conjugated antibody with varying degrees of conjugation occurring at different residues, in agreement with previous studies (Wang et al., 2005; Kim et al., 2014; Goldmacher et al., 2015; Sang et al., 2017). Luo and colleagues previously characterized a Lys antibody drug conjugate observing a drug to antibody ratio ranging from 0–8. In this study, 76 Lys residues were conjugated at different degrees, representing 83% of all putative Lys residues (Luo et al., 2016). For the Nivolumab-Lys, a mixture of species with 28–42 conjugated residues was observed, which implies that potentially all Lys residues might be conjugated to different degrees. The most abundant conjugated forms of Nivolumab-Lys showed 35–36 biotin molecules. Intact LC-MS analysis of the Adalimumab-Lys conjugate showed the presence of a mixture of conjugates containing 2-6 FITC molecules per antibody with the most abundant form containing 2-4 incorporated FITC molecules. This result was in good agreement with the fluorescein incorporation as determined by UV-vis colorimetry. In contrast, the biotin incorporation for Nivolumab determined by HABA/Avidin colorimetric assay was grossly under-estimated, possibly due to a limitation of the assay due to steric effects, which is likely to be a universal limitation of this colorimetric assay. Unexpectedly, it was shown after reduction and LC-MS analysis of Adalimumab-Lys the only indication of fluorescein conjugation was present on the LC.

### HDX-MS Characterization of Cysteine Conjugated Antibodies

The influence of conjugation at Cys residues on the structure of mAbs was determined by HDX-MS experiments as a direct comparison of differential HDX rates between native and conjugated mAbs of both Trastuzumab and Omalizumab (Figure 1). The coverage of the peptide maps at the hinge regions of both antibodies was poor because of the challenges in proteolytic digestion as previously reported (Leurs et al., 2017), however decreased deuterium uptake kinetics were observed for both conjugates Trastuzumab-Cys and Omalizumab-Cys in regions which are in close proximity to the hinge region, when compared with the native forms of the antibodies, Trastuzumab and Omalizumab respectively (Figures 1A,B). This is likely due to the decreased solvent accessibility induced by the conjugation. In contrast, deuterium uptake kinetics are increased in all those mAb regions, which are more distant from conjugation sites, indicating a long-range loss of structure and increase of flexibility induced by the conjugation process including both the reduction and conjugation steps. Particularly, the Trastuzumab-Cys (Figure 1A) showed significantly decreased deuterium uptake kinetics in the C_L_ domain (sequence 206–214) containing Cys214, which interacts with Cys223 of the C_H_1 domain showing decreased deuterium uptake kinetics for adjacent residues 218–220. Increased deuterium uptake kinetics were observed in the regions C_L_ (121–125), C_H_1 (171–177) and C_H_2 (239–255) located distant from the hinge region, consistent with the extension of the typical disulfide bond distance via the incorporated succinimide ring during conjugation, which results in opening up the surrounding protein structure.

The effect of decreased deuterium uptake kinetics in close proximity to conjugation sites was more significant for the Omalizumab-Cys conjugate (Figure 1B) as all four inter-chain disulfide bonds were involved in conjugation, meaning 8 biotin molecules were incorporated in the mAb molecule revealed by LC-MS analysis (Table 1). Furthermore, for the Cys22 in the V_H_ region, decreased deuterium uptake kinetics were observed indicating that this Cys residue maybe more prone to conjugation due to higher steric accessibility. The disulfide bond comprising Cys22 in native Omalizumab might be in the free thiol form as has been shown previously (Harris 2005).

Overall, the conjugation of Omalizumab *via* the classical maleimide linker fluorescein-5-maleimide caused reduction of the disulfide bonds and an increased molecular distance between cysteine residues with a consequent loss of structure and increased flexibility.

### HDX-MS Characterization of Lysine Conjugated Antibodies

The impact of conjugated Lys residues on the mAb structure was determined by HDX-MS as a comparison of differential deuterium uptake kinetics between the native and conjugated Nivolumab and Adalimumab mAbs in the presence and absence of the antigens PD-1 (Nivolumab) and TNFα (Adalimumab) (Figure 2). The Nivolumab-Lys conjugate showed high conjugation at Lys residues with a total label distribution of 28–42 biotin molecules per mAb by intact protein analysis (Table 1). Because of the expected high heterogeneity of conjugated Nivolumab-Lys, it is possible to assume that most of the Lys residues are conjugated to different extents depending on the location of Lys residues within the antibody and accessibility to the conjugation reagents. HDX-MS experiments show that the deuterium uptake kinetics are significantly decreased in the whole Nivolumab-Lys (Figure 2A). It is possible to observe that the Lys residues in the V_H_ region as K43, K57, K65 and K76 show significant decreased deuterium uptake kinetics. Those are in proximity to the CDR regions of HC and the results suggest that conjugation may have an impact on the binding of the antigen for the Optivo-Lys. It has been in fact already reported, that the CDR regions of the HC are predominantly involved in the antigen binding (Lee et al., 2016; Zhang et al., 2020).

The higher order structure of Adalimumab-Lys conjugate with only two FITC molecules on the LC was much less impacted by the conjugation, most likely due to the lower level of conjugation as was observed from the intact mass spectrometry analysis (Figure 2B). In particular, the LC in the F_ab_ region (V_L_, C_L_) shows slightly decreased deuterium uptake kinetics in peptides which contain Lys residues as K42/45 in the N-terminus and K183/188/190 in the C-terminus. Because of the proximity of these Lys residues to each other, it was difficult to determine the site of conjugation without any further experimentation, which was outside the scope of this work. No significant structural differences were observed for the HC of the F_ab_ region (V_H_, C_H_1) when comparing the native and conjugated antibody. However, for CDR-H1 and -H3 increased deuterium uptake kinetics were observed suggesting a loss of structure that may also have an impact on the binding properties of the antibody. The structural differences in the C_H_2 of the Fc domain are similar, albeit on a lesser scale, to the Nivolumab-Lys conjugate indicating that the conjugation has an indirect impact on the mAb structure. According to intact protein MS experiments, the Fc domain was not conjugated.

For the Lys conjugates a correlation between the amount of conjugation and the higher order structural differences observed was clearly demonstrated by HDX-MS experiments (Figure 2). Particularly, an increase in conjugation at Lys residues resulted in a decreased deuterium uptake kinetics across the whole protein structure.

### Characterization of Conjugated mAb Candidates by Different Immunoassay Formats

A panel of mAb conjugates generated were analyzed by different immunoassay formats in order to check differences in the binding properties of the conjugated mAb *vs* the native counterpart. Particularly, seven different assays (Fc binding, complex binding, competitive, immunometric, bridging, Fc capture and streptavidin capture) were initially developed for each pair (conjugated and unconjugated) to discern differences in the binding pattern and provide preliminary information about which part of the mAb, Fab or Fc, has been modified. The different assay formats are explained and summarized in the supporting information S1-4. As previously mentioned, the commercially available antibodies Adalimumab, Nivolumab, Omalizumab and Trastuzumab were conjugated with biotin or FITC using a variety of linkers, chemistries and ratios. The performance of these mAb conjugates regarding modification of the Fc or Fab region was evaluated through comparison of specific assay responses between conjugated and native antibody. The results of the relative Fc binding and antigen (complex) binding of the four selected conjugates Adalimumab-Lys, Nivolumab-Lys, Omalizumab-Cys and Trastuzumab-Cys are listed in Table 2.

The range of recognition values is based on a typical immunoassay CV of ±10%. As recognition values are calculated from two assay results (conjugated, unconjugated), each with CV of 10%, the potential error around the recognition value was calculated as follows: recognition value x 0.818 (0.9/1.1) to recognition value x 1.222 (1.1/0.9).

For the determination of relative Fc recognition of Adalimumab-Lys, Nivolumab-Lys and Omalizumab-Cys, an anti-Fc antibody was used to estimate the differences in the Fc binding between native and conjugated mAb. Similarly, a specific anti-complex mAb for Adalimumab-Lys and Omalizumab-Cys, which detects only the Ab-Ag complex and not Ab or Ag alone, was applied to analyze the relative antigen recognition between native and conjugated mAb. In contrast, the antigen recognition of Nivolumab-Lys was determined by using a competitive assay, where the conjugate binds the immobilized antigen and displacement occurs by varying free antigen concentrations. With increasing concentrations of free antigen, the conjugate is displaced from the immobilized antigen resulting in a signal decrease. Nivolumab conjugates were then ranked by calculating the free antigen concentration required to produce 50% reduction in the signal. In the case of the Trastuzumab-Cys conjugate, the biotin label is used in the streptavidin capture assay to validate the Fc binding and antigen affinity and thus the signals relate to the Trastuzumab conjugate with the least modification of the variable region (100%) out of a mAb conjugate panel.

The relative Fc binding for Adalimumab-Lys is largely unchanged compared to the native mAb showing that the conjugation does not have a significant impact on the Fc domain, whereas the relative antigen recognition is slightly reduced to 91% indicating conjugation might be more present in the Fab region of the mAb. The results for Nivolumab-Lys show a great contrast in the sense that the Fc binding is reduced to 61.1%, whereas the sensitivity in a competitive assay format is actually increased to 132.5% compared to the native mAb. As the Fc binding is significantly reduced due to the conjugation, it is assumed that the conjugation has predominantly modified the Fc region. However, results from the competitive assay indicate some modification of the binding site. Whilst this would typically be expected to reduce the utility of the conjugate, because of the competitive format used, slight changes to antigen recognition appear to have increased the ease with which displacement by free antigen occurs, increasing the sensitivity of the end assay and indicating a change in the Ab-Ag interaction. Trastuzumab-Cys shows the best antigen affinity within a conjugated mAb panel, whereas the Fc binding assay result is significantly reduced to 67% implying that the conjugation has modified mainly the Fc region. Omalizumab-Cys shows a significant reduction in antigen recognition. Whilst there is a detectable reduction in Fc recognition, this is relatively small compared to the change in antigen recognition. Taken together, these results suggest that both Fab and Fc regions of Omalizumab-Cys have been modified, with the Fab modification being most significant in potential assay performance terms.

## Discussion

Monoclonal antibodies are used as analytical tools in immunoassays to immobilize or detect antigens or molecules of interest. In order to do this, conjugation is carried out through a chemical reaction. However, the effects of conjugation regarding amount and location on the structure and functionality of the mAb which ultimately may affect immunoassay performance, are often undetermined. In this study, the conjugation reaction efficiency was studied by intact MS analysis and the impact of conjugation at either Lys or Cys residues on the mAb higher order structure and its binding properties was assessed by HDX-MS and immunoassay respectively. The results of the mass spectrometric characterization of different mAb conjugates correlate very well with the mAb function and binding affinity.

The cysteine conjugation occurs in a specific manner as the inter-chain disulfide bonds are typically targeted. For the Trastuzumab conjugate, the Cys residues of a disulfide bond are dithiosuccinimide-bridged maintaining the disulfide bonding, but with an extension of the typical distance. When using a classical maleimide linker such as fluorescein-5-maleimide for the cysteine conjugation of Omalizumab, the rebuilding of disulfide bridges is not possible. In both cases, the overall mAb structure for Trastuzumab and Omalizumab conjugates show predominantly increased deuterium uptake kinetics due to extended bond distances (Trastuzumab) or reduced disulfide bonds (Omalizumab) resulting in more flexibility in structure. In contrast, protein regions in close proximity to the hinge region and inter-chain disulfide bonds show decreased deuterium uptake kinetics due to the conjugation with bulky groups, which reduces solvent accessibility. The intact protein analysis results correlate well with the HDX-MS results where an increased amount of conjugation per mAb was also associated with a relative decrease in deuterium uptake kinetics. In this regard, the most accessible inter-chain disulfide of Trastuzumab-Cys modified using the chemistry employed is Cys214 of the C_L_ domain and Cys223 of the C_H_1 domain implying no significant structural changes at the antigen binding site. This also correlates well with the immunoassay results that the selected Trastuzumab-Cys mAb shows the best antigen affinity within a conjugated mAb panel. For the Omalizumab-Cys candidate, results of intact protein analysis and HDX-MS reveal that all eight sulfhydryl groups of inter-chain disulfide bonds are conjugated and thus the mAb structure relies only on non-covalent interactions between heavy and light chain causing major structural changes with loss of recognition properties shown by immunoassay experiment results for both the Fab and Fc regions.

As expected, compared to the cysteine conjugation, the conjugation at lysine residues generated more heterogenous mixtures of different conjugated mAb variants (Nivolumab and Adalimumab). Conjugation with biotin and FITC was chemically performed without reduction and therefore the disulfide bridges were maintained. The HDX-MS data showed decreased deuterium uptake kinetics of the conjugated mAbs *vs* the native form, with the relative level of conjugation correlating well with the relative decrease of deuterium uptake kinetics. Particularly, the Nivolumab-Lys is highly conjugated (28–42 biotin molecules per mAb) and the significantly decreased deuterium uptake indicates a potential change of higher order structure that could have a negatively impact on the antigen recognition. This was also confirmed by the immunoassay experiment results. In contrast, conjugation of the Adalimumab mAb had less effects on the deuterium uptake kinetics and binding properties of the antibody as was shown by both HDX-MS and immunoassay experiments. This possibly due to the 2-6 FITC conjugation mostly occurring on the LC as was shown by intact protein MS analysis. Consistent with the immunoassay results which indicate that the conjugation is more present in the Fab region and the Fc part is not significantly impacted.

Overall, this study shows that conjugation can have an impact on the performance of the antibodies used for immunoassay engineering and that mass spectrometry experiments can aid an improved understanding and optimization of the conjugation reaction. This will ultimately aid to better performance. Furthermore, a thorough characterization of the conjugation reaction will ensure continuity in performance over time and across different batches.

## Data Availability

The datasets presented in this study can be found in online repositories. The names of the repository/repositories and accession number(s) can be found in the article/Supplementary Material.
