# Supplementary material for: Impact of Bioconjugation on Structure and Function of Antibodies for Use in Immunoassay by Hydrogen-Deuterium Exchange Mass Spectrometry
Source: Front Mol Biosci. 2022 Jul 7;9:866843. doi: 10.3389/fmolb.2022.866843 (PMC9301968; doi:10.3389/fmolb.2022.866843)
Supplement: Supplementary file 2 [file Table3.docx]

**Supporting Information S1. Impact of Bioconjugation on Structure and Function of Antibodies for Use in Immunoassay by Hydrogen-Deuterium Exchange Mass Spectrometry**

Luise Luckau^1^*, Kate Groves^1^, Chris Blencowe^2^, Sam Scrimshaw^2^, Alastair Dent^2^, Milena Quaglia^1^

^1^National Measurement Laboratory at LGC, Teddington, United Kingdom

^2^Fleet Bioprocessing Ltd, Hartley Wintney, United Kingdom

*Luise.luckau@lgcgroup.com

**S1- 1 Preparation of mAb conjugates**

Concentration of mAbs was determined by UV-vis spectrophotometry using 280 nm extinction coefficients of 1.5 (Trastuzumab, Herceptin), 1.4 (Adalimumab, Humira), 1.7 (Nivolumab, Opdivo) and 1.4 (Omalizumab, Xolair) (mg/mL)^-1^ cm^-1^.

Procedure for the conjugation of Trastuzumab-Cys

Trastuzumab was reconstituted to 21 mg/ml following the supplier instructions and then normalized to 3 mg/ml using TBS pH 7.5 buffer. To Trastuzumab (2.50 mg, 1.67 × 10^‑5^ mmol, 3 mg/ml) was added an aliquot of TCEP.HCl (1.36 mole equivalents, 2.27 × 10^‑5^ mmol, 26 µl, 0.25 mg/ml) in TBS pH 7.5 buffer to target the reduction of a single disulfide per antibody. The mixture was incubated at 20 °C for 90 min. After incubation, the Trastuzumab-SH was desalted by zeba spin desalting column and characterized by UV-vis and Ellman’s colorimetric assay (2.65 mg/ml, Ab:SH = 2.2). To Trastuzumab-SH (2.36 mg, 1.57 × 10^‑5^ mmol, 2.65 mg/ml) was added an aliquot of dibromomaleimide-biotin (4.3 mole equivalents, 6.77 × 10^‑5^ mmol, 7.7 µl, 5 mg/ml) in DMSO. The mixture was incubated at 20 °C for 16 hours. After, an aliquot of the reaction mixture was analyzed by Ellmans colorimetric assay prior to quenching with NEM (5.4 mole equivalents, 7.99 × 10^‑5^ mmol, 40 µl, 0.25 mg/ml) in DMSO to show that 1.9 SH per Ab had reacted. The conjugate was purified by zeba spin desalting column eluting with phosphate pH 7.5, and then analyzed by UV-vis and HABA/Avidin colorimetric assay to afford Trastuzumab (2.2 mg/ml, 80 %, Ab:biotin = 0.9).

Procedure for the conjugation of Omalizumab-Cys

An aliquot of Omalizumab (150 mg/ml) was normalized to 10 mg/ml using phosphate pH 7.5 and then was buffered exchanged into TBS pH 7.5. To Omalizumab (10.5 mg, 7.26 × 10^‑5^ mmol, 5 mg/ml) was added an aliquot of TCEP.HCl (300 mole equivalents, 2.18 × 10^‑2^ mmol, 156 µl, 40 mg/ml) in TBS pH 7.5 buffer to reduce all inter-chain disulfide bonds per antibody. The mixture was incubated at 20 °C for 90 min. After incubation, the Omalizumab-SH was desalted by zeba spin desalting column and characterized by UV-vis and Ellman’s colorimetric assay (3.03 mg/ml, Ab:SH = 7.0). To Omalizumab-SH (9.86 mg, 6.80 × 10^‑5^ mmol, 3.03 mg/ml) was added an aliquot of fluorescein-5-maleimide (32 mole equivalents, 2.18 × 10^‑3^ mmol, 186 µl, 5 mg/ml) in DMSO. The mixture was incubated at 20 °C for 60 min. After, the reaction was stopped by the addition of an aliquot of NEM (5.4 mole equivalents, 7.99 × 10^‑5^ mmol, 40 µl, 0.25 mg/ml) in TBS pH 7.5. The conjugate was purified by zeba spin desalting column eluting with phosphate pH 7.5, and then analyzed by UV-vis to afford Omalizumab‑Cys (2.8 mg/ml, 92 %, Ab:Fluorescein = 5.9).

Procedure for the conjugation of Adalimumab-Lys

An aliquot of Adalimumab (96 mg/ml) was normalized to 10 mg/ml using phosphate pH 7.5 and then was buffered exchanged into phosphate pH 7.5. To Adalimumab (10.0 mg, 6.96 × 10^‑5^ mmol, 5 mg/ml) was added an aliquot of Fluorescein-5-isothiocyanate (FITC) (160 mole equivalents, 1.11 × 10^‑2^ mmol, 108 µl, 40 mg/ml) in DMSO to effect a high Fluorescein incorporation per antibody. The mixture was incubated at 20 °C for 60 min. After, the reaction was stopped by the addition of an aliquot of glycine (800 mole equivalents, 5.57 × 10^‑2^ mmol, 42 µl, 100 mg/ml) in phosphate pH 7.5. The conjugate was purified by zeba spin desalting column eluting with phosphate pH 7.5, and then analyzed by UV-vis to afford Adalimumab-Lys (3.0 mg/ml, 90 %, Ab:Fluorescein = 5.2).

Procedure for the conjugation of Nivolumab-Lys

An aliquot of Nivolumab (10 mg/ml) was buffered exchanged into phosphate pH 7.5. To Nivolumab (9.6 mg, 6.67 × 10^‑5^ mmol, 5 mg/ml) was added an aliquot of Biotin-XX-NHS (80 mole equivalents, 5.34 × 10^‑3^ mmol, 189 µl, 16 mg/ml) in DMSO to effect a high Biotin incorporation per antibody. The mixture was incubated at 20 °C for 60 min. After, the reaction was stopped by the addition of an aliquot of glycine (80 mole equivalents, 2.67 × 10^‑2^ mmol, 20 µl, 100 mg/ml) in phosphate pH 7.5. The conjugate was purified by zeba spin desalting column eluting with phosphate pH 7.5, and then analyzed by UV-vis and HABA/Avidin colorimetric assay to afford Nivolumab-Lys (3.0 mg/ml, 92 %, Ab:Biotin = 16.1).

**S1- 2 Deconvoluted masses of glycosylated and deglycosylated mAbs as measured by LC-MS**

| mAb | m (most abundant glycan) [Da] | m (deglycosylated) [Da] | Most abundant glycan |
| --- | --- | --- | --- |
| Adalimumab | 148,081 | 145,191 | G0F/G0F |
| Nivolumab | 146,221 | 143,331 | G0F/G0F |
| Omalizumab | 149,169 | 146,280 | G0F/G0F |
| Trastuzumab | 148,220 | 145,167 | G0F/G1F |

**S1- 3 Settings for HDX-MS Data Processing**

To generate peptide maps, UDMS^E^ data was analyzed with the ProteinLynx Global Server (PLGS) software v3.0.3 (Waters, Milford, MA) with 165 counts for low energy and 33 counts for high energy. Peak lists were searched against generated protein sequence databases of proteins of interest including pepsin. Amino acid sequences of all proteins are listed below. Peptide and fragment ion tolerances were set to 10 and 20 ppm respectively. In order to identify a protein, at least 2 fragment ions per peptide, 7 fragment ions per protein and 1 peptide per protein should be detected. Enzymatic settings were set to non-specific cleavages and the allowance of 1 missed cleavage. Variable modifications included deamidation of N and Q, oxidation of M, and N-linked glycosylation. The false discovery rate was maintained below 2 %. PLGS output files were imported to DynamX v3.0.0 (Waters, Milford, MA). To generate peptide maps with reproducible identified peptides, peptides must have been observed in at least 4 out of 5 data files (file threshold 4/5), have a minimum number of 0.01 product ions identified per amino acid residue, have a maximum MH+ error of 20 ppm (± 10 ppm) and a maximum allowed relative standard deviation of 1 % for a peptide`s retention time.

**S1- 4 Protein Sequences used in Database Search for PLGS**

Fasta files were created containing the protein sequences of heavy and light chain of each antibody and of the antigens TNFα (UniProt, P01375, 77-233) for Adalimumab and PD-1 (UniProt, Q15116, 24-288) for Nivolumab. To all files, the protein sequence of pepsin (UniProt, P00791, 60-385) was included. Following the amino acid sequences of all antibodies are listed:

Omalizumab (Xolair) heavy chain:

EVQLVESGGGLVQPGGSLRLSCAVSGYSITSGYSWNWIRQAPGKGLEWVASITYDGSTNYADSVKGRFTISRDDSKNTFYLQMNSLRAEDTAVYYCARGSHYFGHWHFAVWGQGTLVTVSSGPSVFPLAPSSKSTSGGTAALGCLVKDYFPEPVTVSWNSGALTSGVHTFPAVLQSSGLYSLSSVVTVPSSSLGTQTYICNVNHKPSNTKVDKKAEPKSCDKTHTCPPCPAPELLGGPSVFLFPPKPKDTLMISRTPEVTCVVVDVSHEDPEVKFNWYVDGVEVHNAKTKPREEQYNSTYRVVSVLTVLHQDWLNGKEYKCKVSNKALPAPIEKTISKAKGQPREPQVYTLPPSRDELTKNQVSLTCLVKGFYPSDIAVEWESNGQPENNYKTTPPVLDSDGSFFLYSKLTVDKSRWQQGNVFSCSVMHEALHNHYTQKSLSLSPGK

Omalizumab (Xolair) light chain:

DIQLTQSPSSLSASVGDRVTITCRASQSVDYDGDSYMNWYQQKPGKAPKLLIYAASYLESGVPSRFSGSGSGTDFTLTISSLQPEDFATYYCQQSHEDPYTFGQGTKVEIKRTVAAPSVFIFPPSDEQLKSGTASVVCLLNNFYPREAKVQWKVDNALQSGNSQESVTEQDSKDSTYSLSSTLTLSKADYEKHKVYACEVTHQGLSSPVTKSFNR

Trastuzumab (Herceptin) heavy chain:

EVQLVESGGGLVQPGGSLRLSCAASGFNIKDTYIHWVRQAPGKGLEWVARIYPTNGYTRYADSVKGRFTISADTSKNTAYLQMNSLRAEDTAVYYCSRWGGDGFYAMDYWGQGTLVTVSSASTKGPSVFPLAPSSKSTSGGTAALGCLVKDYFPEPVTVSWNSGALTSGVHTFPAVLQSSGLYSLSSVVTVPSSSLGTQTYICNVNHKPSNTKVDKKVEPKSCDKTHTCPPCPAPELLGGPSVFLFPPKPKDTLMISRTPEVTCVVVDVSHEDPEVKFNWYVDGVEVHNAKTKPREEQYNSTYRVVSVLTVLHQDWLNGKEYKCKVSNKALPAPIEKTISKAKGQPREPQVYTLPPSREEMTKNQVSLTCLVKGFYPSDIAVEWESNGQPENNYKTTPPVLDSDGSFFLYSKLTVDKSRWQQGNVFSCSVMHEALHNHYTQKSLSLSPGK

Trastuzumab (Herceptin) light chain:

DIQMTQSPSSLSASVGDRVTITCRASQDVNTAVAWYQQKPGKAPKLLIYSASFLYSGVPSRFSGSRSGTDFTLTISSLQPEDFATYYCQQHYTTPPTFGQGTKVEIKRTVAAPSVFIFPPSDEQLKSGTASVVCLLNNFYPREAKVQWKVDNALQSGNSQESVTEQDSKDSTYSLSSTLTLSKADYEKHKVYACEVTHQGLSSPVTKSFNRGEC

Nivolumab (Opdivo) heavy chain:

QVQLVESGGGVVQPGRSLRLDCKASGITFSNSGMHWVRQAPGKGLEWVAVIWYDGSKRYYADSVKGRFTISRDNSKNTLFLQMNSLRAEDTAVYYCATNDDYWGQGTLVTVSSASTKGPSVFPLAPCSRSTSESTAALGCLVKDYFPEPVTVSWNSGALTSGVHTFPAVLQSSGLYSLSSVVTVPSSSLGTKTYTCNVDHKPSNTKVDKRVESKYGPPCPPCPAPEFLGGPSVFLFPPKPKDTLMISRTPEVTCVVVDVSQEDPEVQFNWYVDGVEVHNAKTKPREEQFNSTYRVVSVLTVLHQDWLNGKEYKCKVSNKGLPSSIEKTISKAKGQPREPQVYTLPPSQEEMTKNQVSLTCLVKGFYPSDIAVEWESNGQPENNYKTTPPVLDSDGSFFLYSRLTVDKSRWQEGNVFSCSVMHEALHNHYTQKSLSLSLGK

Nivolumab (Opdivo) light chain:

EIVLTQSPATLSLSPGERATLSCRASQSVSSYLAWYQQKPGQAPRLLIYDASNRATGIPARFSGSGSGTDFTLTISSLEPEDFAVYYCQQSSNWPRTFGQGTKVEIKRTVAAPSVFIFPPSDEQLKSGTASVVCLLNNFYPREAKVQWKVDNALQSGNSQESVTEQDSKDSTYSLSSTLTLSKADYEKHKVYACEVTHQGLSSPVTKSFNRGEC

Adalimumab (Humira) heavy chain:

EVQLVESGGGLVQPGRSLRLSCAASGFTFDDYAMHWVRQAPGKGLEWVSAITWNSGHIDYADSVEGRFTISRDNAKNSLYLQMNSLRAEDTAVYYCAKVSYLSTASSLDYWGQGTLVTVSSASTKGPSVFPLAPSSKSTSGGTAALGCLVKDYFPEPVTVSWNSGALTSGVHTFPAVLQSSGLYSLSSVVTVPSSSLGTQTYICNVNHKPSNTKVDKKVEPKSCDKTHTCPPCPAPELLGGPSVFLFPPKPKDTLMISRTPEVTCVVVDVSHEDPEVKFNWYVDGVEVHNAKTKPREEQYNSTYRVVSVLTVLHQDWLNGKEYKCKVSNKALPAPIEKTISKAKGQPREPQVYTLPPSRDELTKNQVSLTCLVKGFYPSDIAVEWESNGQPENNYKTTPPVLDSDGSFFLYSKLTVDKSRWQQGNVFSCSVMHEALHNHYTQKSLSLSPGK

Adalimumab (Humira) light chain:

DIQMTQSPSSLSASVGDRVTITCRASQGIRNYLAWYQQKPGKAPKLLIYAASTLQSGVPSRFSGSGSGTDFTLTISSLQPEDVATYYCQRYNRAPYTFGQGTKVEIKRTVAAPSVFIFPPSDEQLKSGTASVVCLLNNFYPREAKVQWKVDNALQSGNSQESVTEQDSKDSTYSLSSTLTLSKADYEKHKVYACEVTHQGLSSPVTKSFNRGEC

**S1- 5 Description of immunoassay formats**

| Assay | Format | Used to screen | Information provided |
| --- | --- | --- | --- |
| Fc binding | Immobilized antigen, Conjugate allowed to bind, Binding detected using anti-Fc and quantified relative to unconjugated mAb | All conjugates except Trastuzumab, Label not involved in generating signal | Reduction in signal compared to unconjugated indicates either:   - Reduced recognition of antigen due to modification of Fab region - Reduced binding by anti-Fc due to modification of Fc region |
| Complex binding | Immobilized antigen, Conjugate allowed to bind, Binding detected using anti-complex mAb which recognizes Ag-Ab but not Ag or Ab alone, Quantified relative to unconjugated mAb | All conjugates except Nivolumab and Trastuzumab, Label not involved in generating signal | Reduction in signal compared to unconjugated indicates reduced antigen recognition due to modification of Fab region |
| Competitive | Immobilized antigen, Conjugate allowed to bind in the presence of free antigen, Binding detected using anti-Fc, Concentration of free antigen required to produce 50% displacement of mAb quantified and compared to unconjugated mAb, Maximum signal at Ag zero concentration compared to unconjugated mAb | All conjugates except Trastuzumab, Label not involved in generating signal | Model assay in format used for certain “real world” analytes  Difficult to interpret as increased sensitivity, signaled by a reduced concentration required to produce 50% displacement, could indicate a reduced affinity for antigen, making displacement easier |
| Immunometric | Biotin-mAb conjugate immobilized via streptavidin, allowed to bind free Ag, Binding detected using anti-complex mAb which recognizes Ag-Ab but not Ag or Ab alone, Conjugates ranked by Ag concentration required to produce signal/noise >3 | Omalizumab and Adalimumab biotin conjugates only, Label required to generate signal | Model assay in format used for most “real world” analytes  Difficult to interpret as label required to generate signal  A low incorporation conjugate may show unchanged recognition of Ag compared to unconjugated, but have insufficient label and so be immobilized poorly as a capture surface |
| Bridging | Biotin-mAb conjugate immobilized via streptavidin, allowed to bind free anti-nivolumab, Binding detected using HRP labelled Nivolumab which binds to second arm of captured anti-nivolumab, Conjugates ranked by Ag concentration required to produce signal/noise >3 | Nivolumab biotin conjugates only, Label required to generate signal | Model assay in format used for certain “real world” analytes  Difficult to interpret as label required to generate signal  A low incorporation conjugate may show unchanged recognition of Ag compared to unconjugated, but have insufficient label and so be immobilized poorly as a capture surface |
| Fc capture | Conjugate captured by immobilized anti-Fc, Capture detected with neutralizing anti-trastuzumab which recognizes binding site, Quantified relative to highest signal conjugate | Trastuzumab conjugates only, Label not required to generate signal | Label not required to generate signal  Reduction in signal indicates either:   - Reduced recognition by neutralizing Ab due to modification of Ag binding site - Reduced capture of conjugate due modification of Fc region |
| Streptavidin capture | Biotin-mAb conjugate immobilized via streptavidin, Capture detected with anti-idiotypic trastuzumab which recognizes binding site, Quantified relative to highest signal conjugate | Trastuzumab biotin conjugates only, Label required to generate signal | Label required to generate signal  Reduction in signal indicates either:   - Reduced recognition by anti-idiotypic Ab due to modification of Ag binding site - Reduced capture of conjugate due to low incorporation of biotin |

**Fc binding assay**

- Fixed concentration of the respective antigen in carbonate/bicarbonate coating buffer (pH 9.6) dispensed to wells of polystyrene microtitre plate.
- Plate incubated for 1 hour at 37 °C before well contents aspirated and plate washed three times with PBS T20.
- Plate blocked with BSA solution in PBS T20. After 10 minutes, BSA solution aspirated.
- Fixed concentration of test conjugate or unconjugated control in assay buffer (PBS with BSA) added to coated wells.
- Plate incubated for 1 hour at 37 °C before well contents aspirated and plate washed three times with PBS T20.
- Fixed concentration of anti-Fc labelled with horseradish peroxidase in assay buffer added to wells.
- Plate incubated for 1 hour at 37 °C before well contents aspirated and plate washed three times with PBS T20.
- Tetramethylbenzidine substrate solution dispensed to all wells and colorimetric signal developed at ambient temperature in the dark.
- Colour development stopped by acidification and plate read spectrophotometrically at 450 nm.


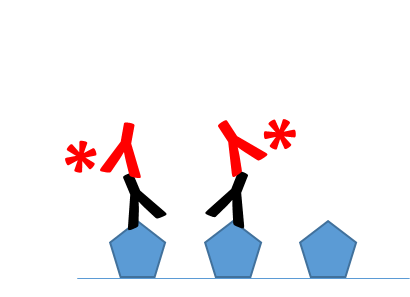


HRP labelled anti-Fc

Test conjugate/control mAb

Immobilised antigen

**Complex binding assay**

- Fixed concentration of the respective antigen in carbonate/bicarbonate coating buffer (pH 9.6) dispensed to wells of polystyrene microtitre plate.
- Plate incubated for 1 hour at 37 °C before well contents aspirated and plate washed three times with PBS T20.
- Plate blocked with BSA solution in PBS T20. After 10 minutes, BSA solution aspirated.
- Fixed concentration of test conjugate or unconjugated control in assay buffer (PBS with BSA) added to coated wells.
- Plate incubated for 1 hour at 37 °C before well contents aspirated and plate washed three times with PBS T20.
- Fixed concentration of anti-complex labelled with horseradish peroxidase in assay buffer added to wells.
  - Anti-complex antibody recognises the respective mAb bound to antigen but not antibody or antigen alone
- Plate incubated for 1 hour at 37 °C before well contents aspirated and plate washed three times with PBS T20.
- Tetramethylbenzidine substrate solution dispensed to all wells and colorimetric signal developed at ambient temperature in the dark.
- Colour development stopped by acidification and plate read spectrophotometrically at 450 nm.


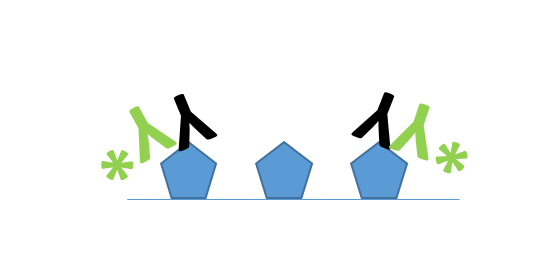


Test conjugate/control mAb

HRP labelled anti-complex

Immobilised antigen

**Competitive assay**

- Fixed concentration of the respective antigen in carbonate/bicarbonate coating buffer (pH 9.6) dispensed to wells of polystyrene microtitre plate.
- Plate incubated for 1 hour at 37 °C before well contents aspirated and plate washed three times with PBS T20.
- Plate blocked with BSA solution in PBS T20. After 10 minutes, BSA solution aspirated.
- Fixed concentration of test conjugate or unconjugated control in assay buffer (PBS with BSA) added to coated wells.
- Varying concentration of free antigen added to each well.
- Plate incubated for 1 hour at 37 °C before well contents aspirated and plate washed three times with PBS T20.
- Fixed concentration of anti-Fc labelled with horseradish peroxidase in assay buffer added to wells.
- Plate incubated for 1 hour at 37 °C before well contents aspirated and plate washed three times with PBS T20.
- Tetramethylbenzidine substrate solution dispensed to all wells and colorimetric signal developed at ambient temperature in the dark.
-
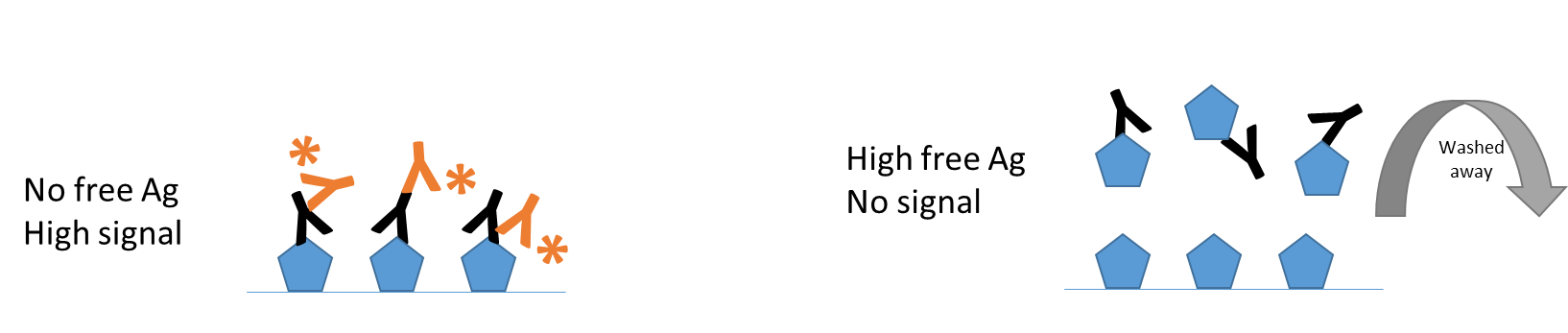
Colour development stopped by acidification and plate read spectrophotometrically at 450 nm.

**Immunometric assay**

- Fixed concentration of test biotin conjugate in assay buffer (PBS with BSA) added to streptavidin coated wells.
- Plate incubated for 1 hour at 37 °C before well contents aspirated and plate washed three times with PBS T20.
- Varying concentration of free antigen added to each well.
- Plate incubated for 1 hour at 37 °C before well contents aspirated and plate washed three times with PBS T20.
- Fixed concentration of anti-complex labelled with horseradish peroxidase in assay buffer added to wells.
- Plate incubated for 1 hour at 37 °C before well contents aspirated and plate washed three times with PBS T20.
- Tetramethylbenzidine substrate solution dispensed to all wells and colorimetric signal developed at ambient temperature in the dark.
- Colour development stopped by acidification and plate read spectrophotometrically at 450 nm.


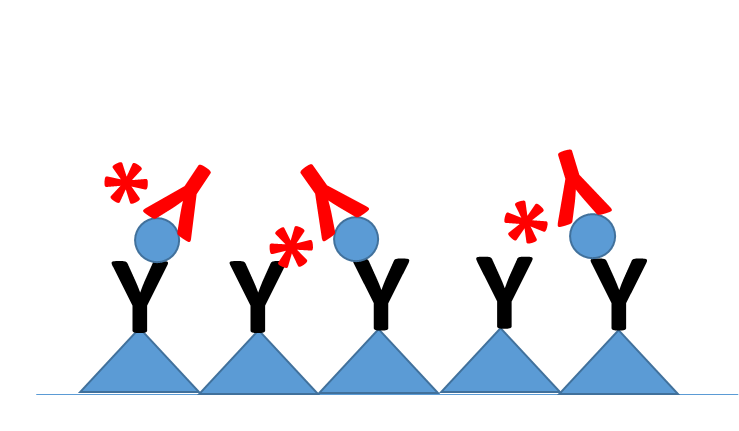


HRP labelled anti-complex

Antigen

Biotin mAb

Immobilised streptavidin

**Bridging assay**

- Fixed concentration of test biotin conjugate in assay buffer (PBS with BSA) added to streptavidin coated wells.
- Plate incubated for 1 hour at 37 °C before well contents aspirated and plate washed three times with PBS T20.
- Varying concentration of anti-nivolumab added to each well.
- Plate incubated for 1 hour at 37 °C before well contents aspirated and plate washed three times with PBS T20.
- Fixed concentration of nivolumab labelled with horseradish peroxidase in assay buffer added to wells.
- Plate incubated for 1 hour at 37 °C before well contents aspirated and plate washed three times with PBS T20.
- Tetramethylbenzidine substrate solution dispensed to all wells and colorimetric signal developed at ambient temperature in the dark.
- Colour development stopped by acidification and plate read spectrophotometrically at 450 nm.

HRP labelled nivolumab

Anti-nivolumab

Biotin nivolumab

Immobilised streptavidin


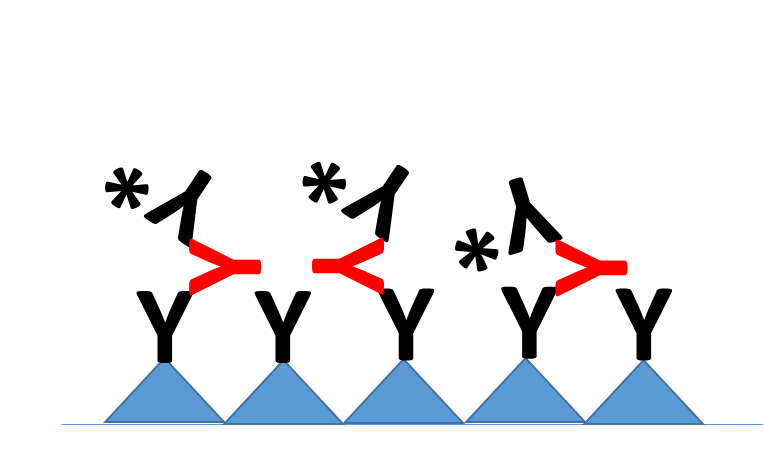


**Fc capture assay**

Assay performed on Gyrolab XP system

Bioaffy 1000 CD

1000-3W-006-A

Method description:

Bioaffy 1000 CD 3-step (C-A-D) wizard run. Analyte spin 2 nl/s Two wash solutions used for needle washes.

Initial needle wash 2 column washes Capture addition 2 column washes Analyte addition 2 column washes Background detect Detection addition 4 column washes Detect 1 %, 5 %, 25 % Final needle wash

- Fixed concentration of biotin labelled anti-Fc conjugate in PBS T20 passed over streptavidin column.
- Fixed concentration of test conjugate passed over streptavidin column
- Fixed concentration of anti-trastuzumab labelled with AlexaFluor 647 passed over streptavidin column
- Fluorescent readout


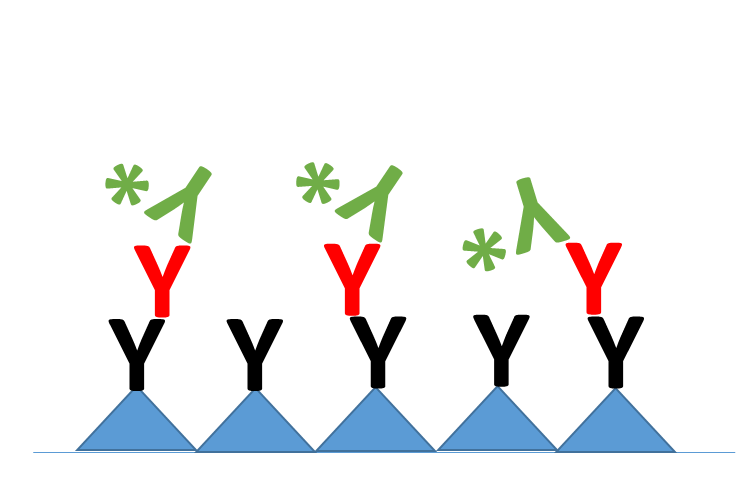


Anti-trastuzumab

Test conjugate

Biotin anti-Fc

Immobilised streptavidin

**Streptavidin capture assay**

Assay performed on Gyrolab XP system

Bioaffy 1000 CD

1000-2W-004-A

Method description:

Bioaffy 1000 CD 2-step (CA-D) wizard run. Analyte spin 2 nl/s Two wash solutions used for needle washes.

Initial needle wash 2 column washes Capture addition 2 column washes Background detect Detection addition 4 column washes Detect 1 %, 5 %, 25 % Final needle wash

- Fixed concentration of test biotin conjugate in PBS T20 passed over streptavidin column.
- Fixed concentration of anti-trastuzumab labelled with AlexaFluor 647 passed over streptavidin column
- Fluorescent readout


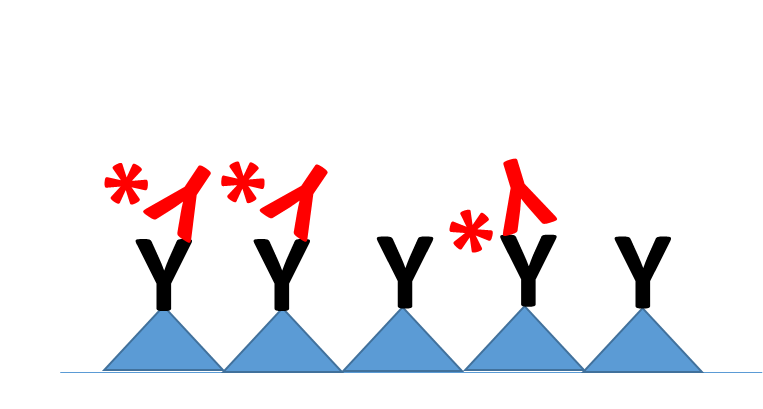


Labelled anti-trastuzumab

Immobilised streptavidin

Biotin Herceptin

**S1- 6 Determination of the amount of conjugation by LC-MS**


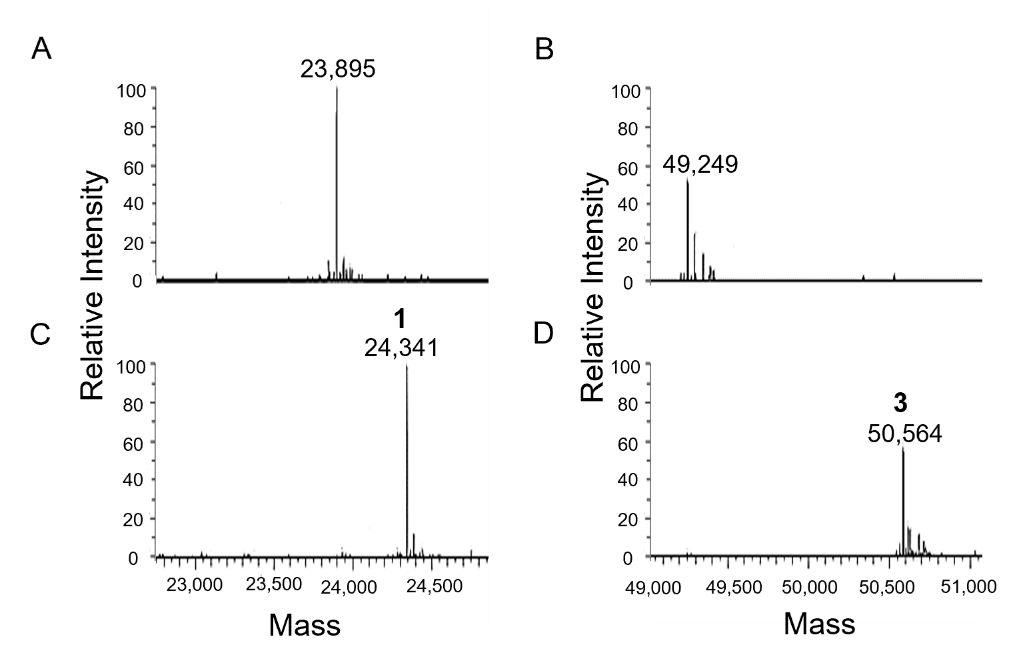


**Figure 1:** Deconvoluted mass spectra of Omalizumab-Cys conjugate: Masses are shown for native LC (A) and HC (B), and for conjugated LC (C) and HC (D). Intact mass of conjugated mAb could not be identified. Bold numbers represent the amount of conjugation with biotin molecules.


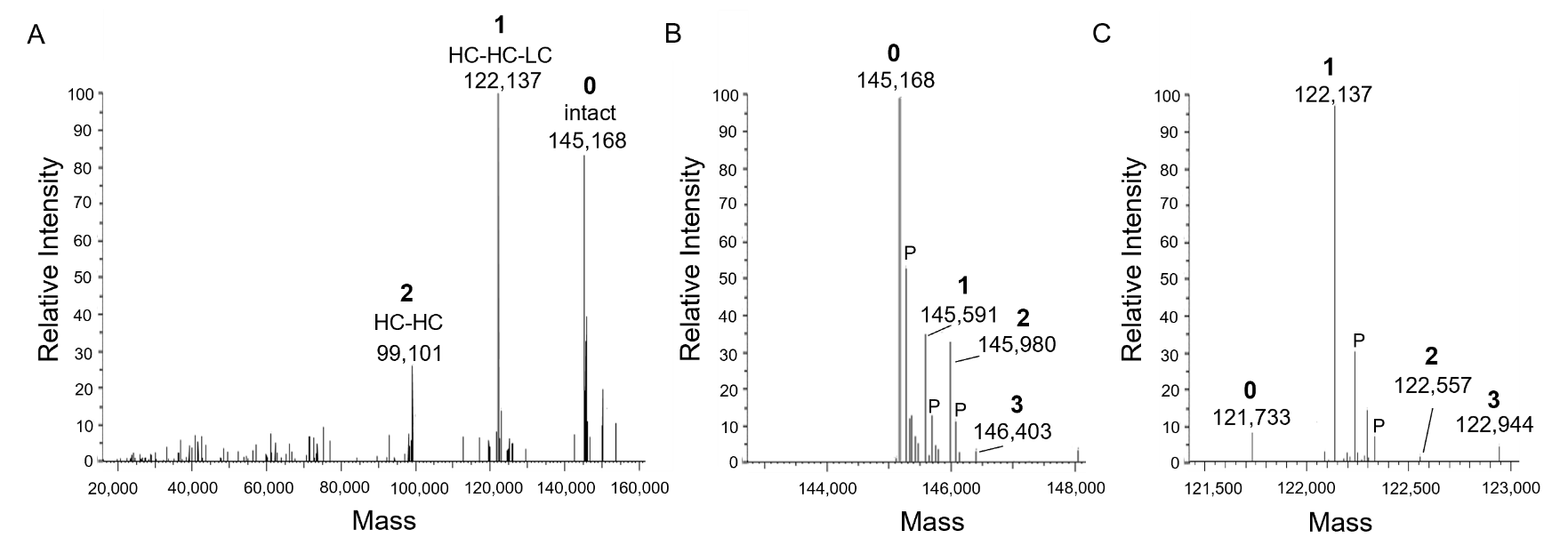


**Figure 2:** Deconvoluted mass spectra of Trastuzumab-Cys conjugate sample with DBM-cross-linked mAb forms (A) and zoomed in spectra of intact mAb masses (B) and HC-HC-LC form (C). Bold numbers represent the amount of conjugation with biotin molecules. Phosphate adducts are highlighted as P.


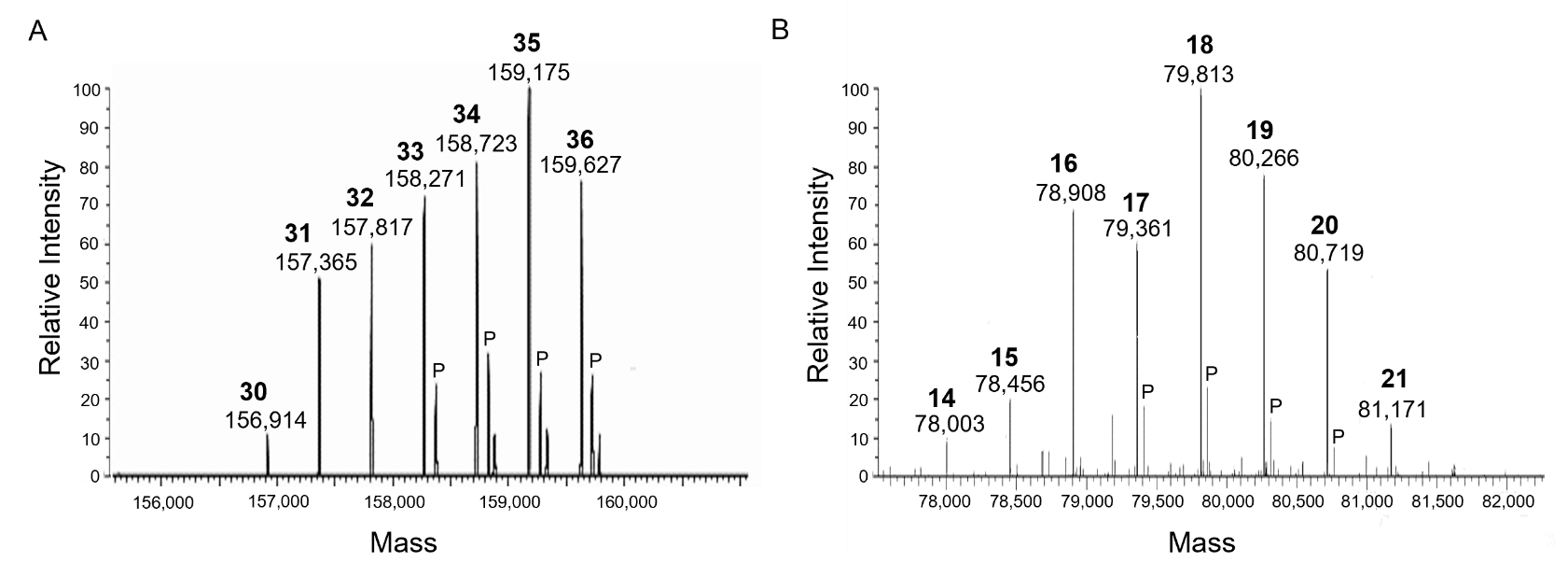


**Figure 3:** Deconvoluted mass spectra of Nivolumab-Lys conjugate: Mass distribution of conjugated intact mAb (A) and HC-LC form (B). Bold numbers represent the amount of conjugation with biotin molecules. Phosphate adducts are highlighted as P.


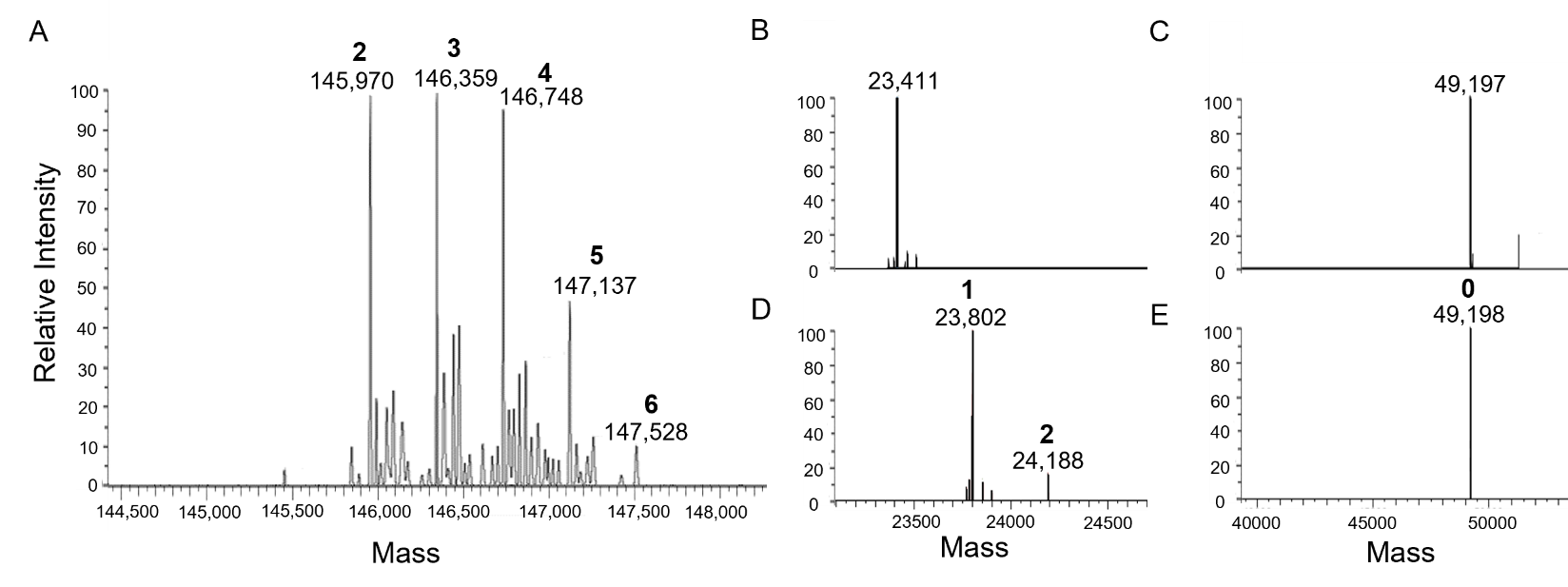


**Figure 4:** Deconvoluted mass spectra of Adalimumab-Lys conjugate: Mass distribution of conjugated intact mAb (A), native LC (B) and HC (C) and conjugated LC (D) and HC (E). Bold numbers represent the amount of conjugation with FITC molecules.

**Table 1:** Conjugation efficiency and amount of Trastuzumab conjugated by using a dibromomaleimide (DBM) derived biotin reagent

| **n (DBM label)** | **m (mAb forms) [Da]** | | **Intensity (mAb forms)** | | **Sum of intensities** | **Relative Abundancy of DBM label amount [%] *** |
| --- | --- | --- | --- | --- | --- | --- |
|  | **intact** | **HC-HC-LC** | **intact** | **HC-HC-LC** | **intact + HC-HC-LC** |  |
| **0** | 145,168 | 121,733 | 2.87E+08 | 1.13E+07 | 2.98E+08 | 46.0 |
| **1** | 145,591 | 122,137 | 1.02E+08 | 1.36E+08 | 2.38E+08 | 36.7 |
| **2** | 145,980 | 122,557 | 9.58E+07 | 1.99E+06 | 9.78E+07 | 15.1 |
| **3** | 146,403 | 122,944 | 8.90E+06 | 5.86E+06 | 1.48E+07 | 02.3 |

*Calculation: Sum of intensities (intact + HC-HC-LC) divided by Sum of all intensities (6.49E+08)

**Table 2:** Summary of mass and conjugation amount determination for mAb conjugates Adalimumab-Lys, Nivolumab-Lys, Omalizumab-Cys and Trastuzumab-Cys

| **mAb** | **m (mAb) [Da]** | | **Δm (mAb) [Da]** | **n (label per mAb)** | **m (LC) [Da]** | | **Δm (LC) [Da]** | **n (label per LC)** | **m (HC) [Da]** | | **Δm (HC) [Da]** | **n (label per HC)** |
| --- | --- | --- | --- | --- | --- | --- | --- | --- | --- | --- | --- | --- |
|  | Control | Conjugate | Conjugate-Control |  | Control | Conjugate | Conjugate-Control |  | Control | Conjugate | Conjugate-Control |  |
| **Adalimumab (Humira)** | 145,191 | 145,970  146,359  146,748  147,137  147,528 | 00,779  01,168  01,557  01,945  02,336 | 02 FITC  03 FITC  04 FITC  05 FITC  06 FITC | 23,411 | 23,802  24,188 | 0390  0777 | 1 FITC  2 FITC | 49,197 | 49,198 | 00000 | 00 FITC |
| **Nivolumab (Opdivo)** | 143,331 | 156,914  157,365  157,817  158,271  158,723  159,175  159,627 | 13,583  14,034  14,486  14,940  15,392  15,844  16,296 | 30 Biotin  31 Biotin  32 Biotin  33 Biotin  34 Biotin  35 Biotin  36 Biotin | 24,314 | na | na | na | 47,350  71,664* | na  78,003*  78,456*  78,908*  79,361*  79,813*  80,266*  80,719*  81,171* | na  6,339*  6,792*  7,244*  7,697*  8,149*  8,602*  9,055*  9,507* | na  14 Biotin*  15 Biotin*  16 Biotin*  17 Biotin*  18 Biotin*  19 Biotin*  20 Biotin*  21 Biotin* |
| **Omalizumab (Xolair)** | 146,280 | na | na | na | 23,895 | 24,341 | 0446 | 1 Fluor | 49,245 | 50,586 | 1,340 | 03 Fluor |
| **Trastuzumab (Herceptin)** | 145,167  121,733 | 145,168  145,591  145,980  146,403  121,733  122,137  122,557  122,944 | 00000  00423  00812  ,1,235  00000  00404  00824  ,1,211 | 00 DBM  01 DBM  02 DBM  03 DBM  00 DBM  01 DBM  02 DBM  03 DBM | 23,439 | na | na | na |  | na | na | na |

FITC: 390 Da, Biotin-XX-NHS: 453 Da, Fluor-5-mal: 446 Da, DBM-Biotin: 406 Da

*mass or conjugation amount per HC-LC [Da]
